# Delayed Presentation of Subclavian Artery Pseudoaneurysm Following Blunt Thoracic Trauma: A Case Report

**DOI:** 10.5811/cpcem.47181

**Published:** 2025-10-22

**Authors:** Matthew E Mollman, Lauren Mays

**Affiliations:** Saint Louis University School of Medicine, Department of Surgery, Division of Emergency Medicine, St. Louis, Missouri

**Keywords:** subclavian artery pseudoaneurysm, point-of-care ultrasound, computed tomography angiography, blunt trauma, case report

## Abstract

**Introduction:**

Subclavian artery pseudoaneurysms are a rare complication of blunt thoracic trauma with high mortality and incidence of long-term disability.

**Case Report:**

We describe a 49-year-old female who suffered a midshaft clavicle fracture after a motorcycle collision who presented five weeks later with right arm weakness, paresthesias, and persistent clavicle pain and swelling. She was diagnosed with a subclavian artery pseudoaneurysm on point-of-care ultrasound performed in the emergency department, which was confirmed with computed tomography angiography. She underwent endovascular stenting but continued to suffer from long-term neurologic deficits related to her condition.

**Conclusion:**

This case underscores that the diagnosis of subclavian artery pseudoaneurysm requires a high index of suspicion. In addition, the case also highlights the utility of point-of-care ultrasound as a modality that can assist in arriving at the diagnosis.

## INTRODUCTION

Traumatic injuries account for approximately 17% of all emergency department (ED) visits annually and are the third leading cause of death when combined for all ages.^1^,^2^ It is estimated that up to 12.8% of these patients suffer a post-traumatic complication, representing significant morbidity.^3^ Subclavian artery pseudoaneurysm is rare, occurring in approximately 1 in 12,500 admitted trauma patients.^4^ This injury is more commonly a result of penetrating trauma; only 25% are secondary to blunt trauma.^5^ Despite successful treatment of the subclavian artery pseudoaneurysm, 50% of patients will continue to experience long-term neurologic deficits.^6^ The diagnosis of SAP is most often made either intra-operatively or with computed tomography angiography; rarely is it first discovered on ultrasound. We report the case of a blunt traumatic subclavian artery pseudoaneurysm with delayed presentation that was initially identified by point-of-care ultrasound (POCUS) in the ED.

## CASE REPORT

A 49-year-old female with a history of polysubstance use disorder, including intravenous heroin, presented to the ED five weeks after a motorcycle accident where she had sustained a direct blow to her right shoulder. She complained of right upper extremity weakness, numbness, and pain. She also noted swelling around her right clavicle. She was not seen at the time of the accident; however, she presented to an outside ED one week after the injury. At the outside facility, radiographs were performed of her chest and shoulder, and she was diagnosed with right 4^th^–8^th^ rib fractures and a midshaft right clavicle fracture. No other imaging was performed at that time, and she was discharged with a sling and provided pain medication. When she presented to our ED for ongoing pain in her upper chest/right shoulder with radiation down the arm and progressive swelling over the clavicle, she also had weakness to her right upper extremity and tingling in her fingers. She denied any subsequent trauma but admitted to not wearing her sling as instructed.

On examination, the patient appeared underweight but in no acute distress. There was tenderness and swelling over the right mid clavicle without overlying skin changes. The right upper extremity showed no deformities or external signs of injury and had tenderness to palpation from the upper arm down through the hand. Pulses were 2+ and symmetric in the radial and ulnar arteries bilaterally, but capillary refill was delayed in the right hand compared to the left. Wrist flexion and extension and hand grip strength were 4/5 on the right compared to 5/5 on the left, and sensation to light touch along the right posterior forearm, palm, and palmar aspect of all five digits and fingertips was diminished. Radiographs were repeated and redemonstrated her previously identified fractures.

**Image 1:**
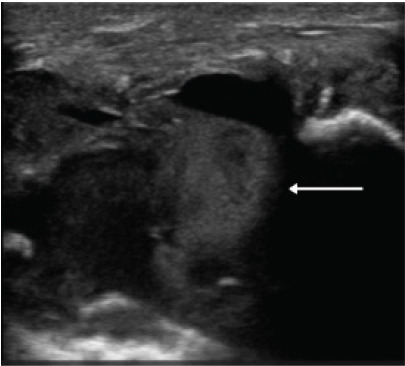
Ultrasound short-axis view of right subclavian artery pseudoaneurysm (arrow).

**Image 2:**
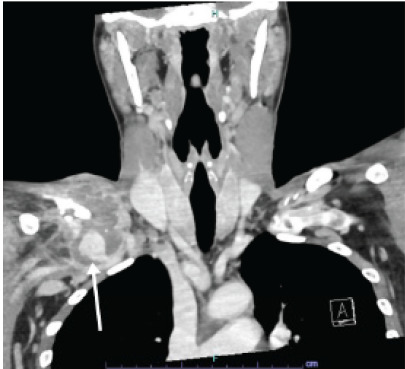
Computed tomography with contrast coronal view of right subclavian artery pseudoaneurysm (arrow).

A POCUS of the clavicle swelling revealed a circular mass inferior to the clavicle with surrounding Doppler flow, raising the likelihood of a vascular etiology (Image 1). Computed tomography angiography (CTA) of the chest and neck confirmed and better characterized the pseudoaneurysm: a 5.5-cm partially thrombosed right subclavian artery pseudoaneurysm with extension above the clavicle (Image 2).

After consultation with vascular surgery, the patient underwent endovascular repair with stent placement. Postoperatively, she reported improvement in her paresthesias and regained full strength in her right arm and hand. The patient was discharged the following day on dual-antiplatelet therapy with aspirin and clopidogrel.

*CPC-EM Capsule*

What do we already know about this clinical entity?

*Traumatic subclavian pseudoaneurysms are potentially life-threatening injuries that may cause long-term neurovascular complications*.

What makes this presentation of disease reportable?

*The delayed presentation in this case with soft findings of vascular injury highlights the importance of maintaining a high level of suspicion*.

What is the major learning point?

*Point-of-care ultrasound is a quick, non-invasive diagnostic tool that can aid in the detection of a traumatic subclavian pseudoaneurysm*.

How might this improve emergency medicine practice?

*This case highlights a rare but serious complication of a common thoracic injury and the use of point-of-care ultrasound to aid in the diagnosis*.

## DISCUSSION

We present a rare case of a blunt trauma subclavian pseudoaneurysm with delayed presentation. The diagnosis of a subclavian pseudoaneurysm is time sensitive due to the risk of rupture that may result in catastrophic hemorrhage and death. Rupture and hemorrhage occurs in approximately 10% of untreated subclavian artery pseudoaneurysms.^7^ Other potential complications arising from delayed diagnosis include thromboembolic events, limb ischemia, and neurologic deficits.^8^ Although blunt subclavian artery injuries are rare, accounting for only 2% of all blunt vascular injuries, the mortality is high.^8^,^9^ Only about 15% of patients with blunt subclavian artery injuries arrive alive to the hospital, and of those the mortality remains high at up to 30%.^5^,^9^ As with other subclavian artery injuries, mortality from subclavian artery pseudoaneurysms is due to exsanguination. Hard signs of vascular injury such as absent pulses, a rapidly expanding hematoma, palpable thrill, or active pulsatile bleeding are likely to prompt vascular imaging. This imaging often aids in the rapid diagnosis of a subclavian artery injury. However, soft signs such as a peripheral nerve deficit or a small nonpulsatile hematoma over an artery, must be considered as well.

In our case, the patient did not demonstrate hard signs of vascular injury but did have soft signs including a new neurologic deficit in her right upper extremity and a small nonpulsatile hematoma over the site of her arterial injury. The delayed presentation of a subclavian pseudoaneurysm secondary to blunt trauma is even more uncommon and thus requires a high index of suspicion. Presenting symptoms often involve swelling and pain at the site of injury, chest pain and/or referred pain to the ipsilateral shoulder. As in this case, during the patient’s first presentation, those symptoms may be initially falsely attributed solely to bony injuries to the overlying clavicle and rib fractures if radiograph is the only imaging modality used. In blunt chest trauma presenting several days after the injury, clinicians may assume that radiograph imaging is sufficient to identify significant non-bony traumatic injuries of the chest such as pneumothorax, hemothorax, or pulmonary contusions. This case demonstrates the importance of considering less obvious traumatic thoracic injuries, especially in the context of a high-risk mechanism, and obtaining advanced imaging such as CTA.

Other symptoms of a subclavian artery pseudoaneurysm may include upper extremity paresthesias, motor weakness, and even paralysis secondary to either claudication or compression of the brachial plexus. These symptoms could be erroneously attributed to either a cervical spine or cerebral process prompting CT of the head/neck that might fail to diagnose the true pathology. The diagnosis is typically made by CTA of the chest due to its wide availability and accuracy. This case is the first reported use of POCUS to diagnose a subclavian artery pseudoaneurysm in the ED. The findings on POCUS prompted the decision to obtain a CTA of the chest to further evaluate the patient’s symptoms. Ultrasound can be performed immediately at the bedside, is non-invasive, and can serve to expedite further diagnostic studies and subsequent consultation with vascular surgery.

Endovascular repair with stent placement is now the preferred treatment option. Open vascular repair is less favorable as access to the subclavian artery is difficult. Despite successful treatment, many patients suffer long-term neurologic symptoms caused by brachial plexus compression by the pseudoaneurysm.^9^ Nerve damage tends to be worse in those who have a delay in diagnosis and emphasizes the need for prompt diagnosis.

## CONCLUSION

Blunt traumatic subclavian artery pseudoaneurysms carry a high rate of mortality and morbidity and require a high index of suspicion in the setting of significant blunt chest trauma. Point-of-care ultrasound can provide rapid, non-invasive identification of subclavian artery injury and pseudoaneurysm formation. Timely diagnosis is critical to reducing the associated morbidity. Despite treatment, some patients will suffer chronic neurologic sequelae resulting from secondary brachial plexus injury.
